# Platelets, a reliable source for peripheral Alzheimer’s disease biomarkers?

**DOI:** 10.1186/2051-5960-2-65

**Published:** 2014-06-16

**Authors:** Michael Veitinger, Balazs Varga, Sheila B Guterres, Maria Zellner

**Affiliations:** Institute of Physiology, Centre for Physiology and Pharmacology, Medical University of Vienna, Schwarzspanierstrasse 17, 1090 Vienna, EU, Austria; Institute of Chemistry at São Carlos, University of São Paulo, São Paulo, Brazil

**Keywords:** Alzheimer’s disease, Diagnosis, Platelets, Peripheral biomarker, APP, Amyloid beta, Tau, Mao-B, Neurotransmitter, Hedge effect size

## Abstract

Peripheral biomarkers play an indispensable role in quick and reliable diagnoses of any kind of disease. With the population ageing, the number of people suffering from age-related diseases is expected to rise dramatically over the coming decades. In particular, all types of cognitive deficits, such as Alzheimer’s disease, will increase. Alzheimer’s disease is characterised mainly by coexistence of amyloid plaques and neurofibrillary tangles in brain. Reliable identification of such molecular characteristics antemortem, however, is problematic due to restricted availability of appropriate sample material and definitive diagnosis is only possible postmortem. Currently, the best molecular biomarkers available for antemortem diagnosis originate from cerebrospinal fluid. Though, this is not convenient for routine diagnosis because of the required invasive lumbar puncture. As a consequence, there is a growing demand for additional peripheral biomarkers in a more readily accessible sample material. Blood platelets, due to shared biochemical properties with neurons, can constitute an attractive alternative as discussed here. This review summarises potential platelet Alzheimer’s disease biomarkers, their role, implication, and alteration in the disease. For easy comparison of their performance, the Hedge effect size was calculated whenever data were available.

## Introduction

### Alzheimer’s disease

One of the most common forms of dementia is Alzheimer’s disease (AD), which is characterised by a progressive cognitive decline. It is estimated to currently affect about 35 million people worldwide and by 2050 this number is predicted to increase to >115 million, thus reaching over 1% of the total population. Loss of memory is common amongst the aged and once this condition becomes more pronounced, it is termed ‘amnestic mild cognitive impairment’ (aMCI) and is often considered as a very early stage of AD. Early-onset familiar AD (EOFAD) is caused by genetic factors like mutations in genes encoding the amyloid precursor protein (APP) and the subunits of the APP-cleaving enzyme γ-secretase, termed presenilin-1 and −2 (PS1 and PS2). The present review, however, focuses on the more common late-onset AD (LOAD), which is characterised by more heterogeneous conditions [1]. Besides neurofibrillary tangles (NFT) and plaques composed of amyloid beta (Aβ), typical neuropathological characteristics associated with AD are loss of cholinergic neurons in the basal forebrain and reduced acetylcholine concentrations [2]. In addition, neocortical biopsies of AD brains show a severe decrease in the levels of the three monoamine neurotransmitters dopamine, norepinephrine, and serotonin [3]. Further comorbid signs of AD are changes in the metabolome such as vitamin B_12_ and folate deficiencies, as well as elevated homocysteine levels, potentially providing a functional link to the assumed influence of lifestyle factors on AD development [4].

Despite worldwide efforts at improving AD detection, ‘definite’ AD diagnosis is still restricted to postmortem evaluation of coexisting plaques and NFTs in brain preparations from deceased patients [5]. Antemortem, one of the most reliable diagnostic tools is brain imaging, i.e. visualisation of atrophies most apparent in the temporal lobe [6]. Hippocampal lesions in patients appear to be very important characteristics to determine progression from aMCI to overt AD [7]. However, brain imaging requires exorbitantly expensive equipment and highly trained staff, making these examinations a low-throughput screening method. Therefore, easily accessible molecular markers for early and reliable diagnosis would be of tremendous importance. Moreover, correct diagnosis at the stage of aMCI followed by immediate treatment may be more efficacious than administering medication later on [8, 9].

### Biomarker sources for AD

Cerebrospinal fluid (CSF) Aβ_1–42_, total tau, and phospho-tau_181_ represent some of the best available AD biomarkers for clinical diagnosis. When combined, they give accuracy in the range of 90% [10]. A significant drawback is the required invasive lumbar puncture, making CSF impractical for initial diagnosis or clinical routine screening. Easily accessible blood, in contrast, is a desired sample material and multiple plasma proteins have already been identified as potential AD biomarker candidates, for example cytokines, acute phase proteins, adhesion molecules, and growth factors [11]. Nevertheless, most of these seem to reflect secondary inflammatory processes of neurodegeneration rather than being causative agents. In addition, many factors are likely to influence the abundance of these plasma markers, e.g. accompanying infection and inflammation, as well as disease progression of AD itself. Because of that, many AD-related plasma profiles are hard to reproduce [12]. Since the mean biological variation of platelet proteins is more stable (coefficient of variation, CV = 17% [13]) as compared to other body fluids such as plasma (CV = 23% [14]), urine (CV = 82% [15]), or CSF (CV = 83% [16]) and since AD-related pathological changes responsible for neuron decay most likely start inside cells, cellular biomarkers may be a more preferable choice for detection of causal AD signs.

### Platelets and their implication as peripheral model for neurons

**Figure 1:**
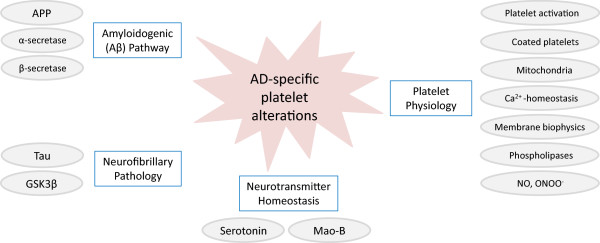
**Overview of potential peripheral platelet AD biomarkers.** Besides key players involved in the generation of amyloid plaques and neurofibrillary tangles, a good many additional AD-specific platelet alterations have been described. APP, amyloid precursor protein; GSK3β, glycogen synthase kinase 3β; Mao-B, monoamine oxidase B; NO, nitric oxide; ONOO^−^, peroxynitrite.

Blood platelets are cell fragments shed from megakaryocytes and released into the blood stream. Therefore, platelets lack a nucleus but contain a number of organelles such as mitochondria for energy generation, dense granules for storage of small molecules (e.g. serotonin, ADP, etc.), and α-granules serving as reservoir for secretory proteins. Upon stimulation, a process required for haemostasis and other physiological functions, platelets undergo a rapid shape change and release a repertoire of substances from mentioned granules [17]. With regards to neurons, they have been described to share many biochemical similarities [18–20] and mirror abnormalities in psychiatric and neuronal disorders [21, 22]. For instance, platelets express high levels of APP [23], in fact highest concentrations of this protein are found in brain and platelets. Additionally, tau protein has recently been detected in their proteome [24, 25]. Both proteins are described to have different isoform patterns in AD [26, 27]. A more obvious advantage of platelets is their fast, easy, and minimally invasive accessibility from whole blood in high numbers [28]. For these reasons, several research teams investigated whether AD-related changes of the brain are also reflected in platelets. This review aims to provide an overview of these studies with particular emphasis on the reliability of potential platelet AD biomarkers summarised in Figure 1. Additionally, in order to enable the comparison of their diagnostic power, we calculated the Hedge effect size (ES) http://www.polyu.edu.hk/mm/effectsizefaqs/calculator/calculator.html [29] for accessible data sets (Table 1). The relative values, either positive or negative (up- or down-regulation), are a measure of the difference of mean values between groups under consideration of their standard deviation. Thus, the higher the ES, the bigger the difference of a parameter with concomitant small standard deviations between the groups analysed, indicating a more powerful biomarker candidate.

**Table 1:** **Comparison of platelet AD biomarker performance by Hedge effect size**

AD platelet biomarkers	Reg. AD	AD			Hedge ES	Controls			Pubmed ID
		*n*	Age	MMSE		MMSE	Age	*n*	
**ADAM 10**	**↓**	10^*a*^	75 ± 8	16 ± 5	**−4.4**	27 ± 2	76 ± 7	8	[30]
**ADAM 10**	**↓**	11^*b*^	76 ± 8	13 ± 5	**−2.6**	27 ± 2	76 ± 7	8	[30]
**ADAM 10**	**↓**	9^*c*^	75 ± 8	1 ± 2	**−2.0**	27 ± 2	76 ± 7	9	[30]
**APP total mRNA**	**↑**	20	78 ± 5	17 ± 4	**1.8**	29 ± 1	76 ± 5	18	[31]
**APP KPI mRNA**	**↑**	20	78 ± 5	17 ± 4	**1.2**	29 ± 1	76 ± 5	18	[31]
**APP 115 kDa protein abundance**	**↑**	30	67 ± 10	25 ± 3	**1.6**	29 ± 1	68 ± 10	23	[32]
**APP ratio 130/106-110 kDa ratio**	**↓**	30	67 ± 10	25 ± 3	**−2.0**	29 ± 1	68 ± 10	23	[32]
**APP ratio 130/106-110 kDa ratio**	**↓**	12	71 ± 2	28 ± 2	**−1.2**	29 ± 2	70 ± 6	10*	[33]
**APP ratio 130/106-110 kDa ratio**	**↓**	33	68 ± 6	18 ± 4	**−2.4**	29 ± 1	63 ± 6	26	[34]
**APP ratio 130/106-110 kDa ratio**	**↓**	32	72 ± 10	13 ± 7	**−3.0**	28 ± 4	68 ± 14	25	[35]
**APP ratio 130/106-110 kDa ratio**	**↓**	85	68 ± 0	14 ± 7	**−2.4**	29 ± 2	n.a.	24	[36]
**APP ratio 130/106-110 kDa ratio**	**↓**	23	74 ± 9	19 ± 5	**−1.1**	29 ± 1	70 ± 6	29	[37]
**APP ratio 130/106-110 kDa ratio**	**↔**	66	77 ± 10	14 ± 8	**−0.1**	29 ± 1	73 ± 11	46	[38]
**APP ratio 130/106-110 kDa ratio**	**↓**	15	n.a	n.a	**−1.0**	n.a	n.a	19	[39]
**APP ratio 130/106-110 kDa ratio (AChE-inhib.)**	**↓**	20	70 ± 10	19 ± 4	**−0.4**	28 ± 2	70 ± 10	10	[40]
**BACE1-β secretase activity**	**↑**	86	80 ± 7	18 ± 5	**0.6**	29 ± 1	79 ± 8	115	[41]
**BACE1-β secretase activity**	**↑**	52*	76	25 ± 1	**0.7**	30	74	75	[42]
**BACE1 whole protein abundance**	**↓**	15	82 ± 5	19 ± 6	**−0.9**	29 ± 1	80 ± 5	12	[43]
**Calcium intracellular**	**↑**	100	68 ± 7	n.a.	**2.0**	n.a.	65 ± 9	50	[44]
**Calcium intracellular female**	**↑**	60	72 ± 7	18 ± 2	**1.6**	29 ± 1	70 ± 8	25	[45]
**Calcium intracellular male**	**↑**	40	66 ± 5	18 ± 3	**1.5**	29 ± 2	63 ± 4	25	[45]
**Coated-platelet levels**	**↑**	10	n.a.	< 20	**1.1**	n.a.	n.a.	19	[46]
**Coated-platelet levels**	**↑**	36*	74 ± 8	28 ± 1	**0.5**	28 ± 1	73 ± 8.8	30*	[47]
**Coated-platelet levels**	**↑**	20	72 ± 10	23 ± 2	**0.9**	30 ± 0	74 ± 7	40	[48]
**Cytochrome c oxidase activity**	**↓**	10	<60	n.a.	**−0.8**	n.a	61 ± 3	5	[49]
**Cytochrome c oxidase activity**	**↓**	10	>60	n.a.	**−1.0**	n.a	61 ± 3	5	[49]
**Cytochrome c oxidase activity**	**↓**	20	65 ± 9	18 ± 5	**−4.4**	n.a.	63 ± 9	20	[50]
**Cytochrome c oxidase activity**	**↓**	22	66 ± 9	17 ± 8	**−1.5**	26 ± 3	63 ± 9	20	[51]
**Cytochrome c oxidase activity**	**↓**	6	n.a.	n.a.	**−1.3**	n.a.	n.a.	8	[52]
**Cytochrome c oxidase activity (Complex IV)**	**↓**	8	78 ± 7	17 ± 7	**−1.4**	30 ± 1	73 ± 5.7	7	[53]
**Cytochrome c oxidase activity (Complex IV)**	**↓**	5*	78 ± 10	26 ± 2	**−1.4**	30 ± 1	73 ± 5.7	7	[53]
**GSK3β Ser-9 phosphorylated/total GSK3β**	**↓**	24	76 ± 4	19 ± 4	**−1.1**	28 ± 3	71 ± 5	23	[54]
**GSK3β Ser-9 phosphorylated/total GSK3β**	**↓**	22*	74 ± 7	26 ± 2	**−0.6**	28 ± 3	71 ± 5	23	[54]
**Hyperacidification after activation**	**↓**	19	71 ± 5	n.a.	**4.7**	n.a.	61 ± 8	14	[55]
**Immunoglobulin**	**↑**	25	78 ± 1	21 ± 1	**2.7**	28 ± 0	71 ± 2	26	[56]
**Mao-B activity (phenylethylamine)**	**↑**	20	81 ± 11	5 ± 7	**1.0**	28 ± 1	80 ± 11	9	[57]
**Mao-B activity (phenylethylamine)**	**↔**	15	68 ± 3	n.a.	**1.2**	n.a.	54 ± 2	8	[58]
**Mao-B activity (benzylamine)**	**↑**	50	68 ± 14	n.a.	**2.1**	n.a.	64 ± 14	50	[59]
**Mao-B activity (phenylethylamine)**	**↑**	11	65 ± 1	n.a.	**1.2**	n.a.	65	11	[60]
**Mao-B activity (kynuramine)**	**↔**	11	64 ± 7	14 ± 4	**−0.7**	n.a.	64 ± 8	11	[61]
**Mao-B protein abundance**	**↑**	34	79 ± 8	5 ± 4	**1.4**	28 ± 2	79 ± 9	34	[62]
**Mean platelet volume**	**↑**	126	76 ± 7	n.a.	**0.3**	n.a.	75 ± 6	286	[63]
**Membrane fluidity**	**↑**	12	72 ± 4	11 ± 2	**1.0**	n.a.	68 ± 4.5	18	[64]
**Membrane fluidity**	**↔**	23	74 ± 9	19 ± 5	**−0.5**	29 ± 1	70 ± 5.8	29	[37]
**Membrane fluidity**	**↑**	24	n.a.	n.a.	**−1.6**	n.a.	n.a.	36	[65]
**Membrane fluidity external**	**↓**	100	68 ± 7	n.a.	**0.3**	n.a.	65 ± 9	50	[44]
**Membrane fluidity in submitoch. particles**	**↓**	30	n.a.	n.a.	**−2.3**	n.a.	n.a.	30	[66]
**Membrane fluidity internal**	**↓**	100	68 ± 7	n.a.	**0.8**	n.a.	65 ± 9	50	[44]
**Na+/K + −ATPase activity**	**↓**	100	68 ± 7	n.a.	**−6.0**	n.a.	65 ± 9	50	[44]
**Na+/K + −ATPase activity female**	**↓**	60	72 ± 7	18 ± 2	**−8.5**	29 ± 1	70 ± 8	25	[45]
**Na+/K + −ATPase activity male**	**↓**	40	66 ± 5	18 ± 3	**−7.3**	29 ± 2	63 ± 4	25	[45]
**NO production**	**↑**	100	68 ± 7	n.a.	**6.3**	n.a.	65 ± 9	50	[44]
**NO production female**	**↑**	60	72 ± 7	18 ± 2	**4.5**	29 ± 1	70 ± 8	25	[45]
**NO production male**	**↑**	40	66 ± 5	18 ± 3	**6.5**	29 ± 2	63 ± 4	25	[45]
**ONOO** ^**−**^ **production**	**↑**	100	68 ± 7	n.a.	**6.8**	n.a.	65 ± 9	50	[44]
**ONOO** ^**−**^ **production female**	**↑**	60	72 ± 7	18 ± 2	**8.4**	29 ± 1	70 ± 8	25	[45]
**ONOO** ^**−**^ **production male**	**↑**	40	66 ± 5	18 ± 3	**8.1**	29 ± 2	63 ± 4	25	[45]
**Phospholipase A2 activity**	**↓**	16	70 ± 11	n.a.	**−0.9**	n.a.	63 ± 10	13	[67]
**Phospholipase A2 activity**	**↓**	21	75 ± 7	14 ± 9	**−1.6**	28 ± 2	73 ± 5	17	[68]
**Phospholipase A2 activity**	**↓**	11*	73 ± 5	25 ± 4	**−0.7**	28 ± 2	73 ± 5	17	[68]
**Phospholipase A2 activity**	**↑**	37	73 ± 6	19 ± 5	**0.3**	n.a.	72 ± 5	27	[69]
**Phospholipase A2 activity**	**↓**	44	75 ± 7	19 ± 5	**−0.6**	28 ± 4	75 ± 7	66	[70]
**Phospholipase A2 activity**	**↓**	59*	72 ± 6	27 ± 3	**−0.6**	28 ± 4	75 ± 7	66	[70]
**Phospholipase C delta protein and activity**	**↓**	10	81 ± 1	1 ± 2	**−3.4**	1 ± 2	80 ± 2	10	[71]
**Serotonin [c]**	**↓**	22	n.a.	n.a.	**−1.5**	n.a.	n.a.	20	[72]
**Serotonin [c]**	**↑**	57	n.a.	n.a.	**1.0**	n.a.	n.a.	20	[73]
**Tau high molecular weight/monomeric tau**	**↑**	15	81	15	**1.6**	28	68	10	[24]
**TRAP-induced CD62P expression [%]**	**↓**	23	70 ± 8	n.a.	**−1.2**	n.a.	60 ± 10	17	[74]

The Hedge effect size was calculated based on published values of mean, standard deviation, standard error of mean, and sample size. Positive ES indicate up-regulation, negative ES down-regulation in AD patients. ^a^Mild AD, ^b^Moderate AD, ^c^Advanced AD, *MCI.

## Review

### Platelet activation

Platelets can become activated by a number of agonists. This introduces shape changes and degranulation of dense- and α-granules [17], redistributing several proteins from α-granules to their surface. An elevated degree of platelet activation in AD patients has been reported by quantification of CD62P (P-selectin) surface expression, platelet aggregates, and platelet leukocytes complexes [75]. Results from another study contradict these findings as these show no difference in surface CD62P expression of unstimulated platelets but significantly lower levels in thrombin receptor activating peptide 6 (TRAP-6)-activated AD platelets [74]. However, this may be a sign of already exhausted platelets *in vivo* after sustained activation, reflected by the increase of sCD62P plasma levels [74].

### Coated platelets

Upon parallel activation with collagen and thrombin, a subset of so called ‘coated’ platelets retains augmented concentrations of pro-coagulant proteins on their cell membrane [76]. Determination of surface fibrinogen levels by flow cytometry is an established method for their quantification [77]. Coated platelet numbers have been found to be significantly increased in early stages of AD as compared to matched controls but decreased in late stages of AD, even below levels of cognitively healthy individuals [48, 78–80]. This group also showed that coated platelets can be used to predict which aMCI patients would rapidly progress to AD [80]. Additionally, this parameter could distinguish amnestic from non-amnestic MCI patients by higher levels of coated platelets in these patients [47]. Similarly, they suggested that these elevated levels could discriminate frontotemporal dementia from AD [48]. Importantly, all these publications originate from the same research group; therefore, and because of the only moderate sample numbers and ES (Table 1), reproduction by other international experts is stringently required.

### Serotonin metabolism

The idea of using platelets as an experimental system for neurons was triggered by discovering that platelets’ dense granules (which release their content after stimulation) are the major storage site for serotonin (5-hydroxytryptamin, 5-HT) in blood [81], similar to its vesicular storage in neurons [82]. Consequently, experiments with the antipsychotic drug reserpine were carried out. Reserpine mediates depletion of monoamine neurotransmitters in synapses; the same effect could be simulated in platelets [18]. Since then, many studies confirmed that platelets are an adequate model resembling storage and release of serotonin from serotonergic neurons under both physiological and pathological conditions [83–85]. Moreover, a significant correlation between interindividual 5-HT_2A_ receptor binding characteristics in the brain cortex and in platelets has been observed both in animals [86] and humans [87]. A decrease in serotonin uptake and in the number of transporter binding sites in brain tissue and platelets has been shown to be general molecular evidence for serotonergic abnormalities in depression [85, 88].

### Serotonin metabolism in platelets of AD patients

Concentrations of serotonin and 5-hydroxyindoleacetic acid, serotonin uptake, and K^+^-stimulated release of endogenous serotonin have all been found to be reduced below control values in neocortical biopsy samples from patients with histologically verified AD [89]. A significant reduction in serotonin binding in non-depressed AD patients hints that presynaptic serotonergic function is already affected before development of psychiatric problems such as depression [90]. Further evidence for reflection of cerebral biochemical abnormalities in platelets was the diminished affinity of the platelet 5-HT_2A_ receptor towards its radioactively labelled ligand [3H] LSD in AD cases [91], suggesting decreased serotonin uptake by these peripheral cell bodies. A number of other studies also registered an impaired uptake of serotonin in platelets [92–94]. However, the reliability of this marker is questionable since this finding could not be reproduced in two other investigations [95, 96]. In strong contrast, another study even showed increased accumulation of the neurotransmitter in platelets of female AD patients [97]. Intraplatelet serotonin concentrations reported in literature are also quite inconsistent as reduced [72, 98, 99], as well as increased [73] values in AD patients with delusions have been measured. In the latter observation, there might be a weak parallel to findings of higher serotonin level in AD patients with psychotic features than in inconspicuous subjects [98]. Similarly, in demented patients concentrations of platelet serotonin were higher as compared to controls [73]. Further details about the role of serotonin in depression, ageing, and AD can be found in the review by Meltzer *et al.* [100] which also compares results from platelets and neurons. Some of the discrepancies may arise from different stages of AD, given a recent study showing that serotonin levels were only reduced in late stage AD [99]. Additionally, diurnal and seasonal variations have been described for intraplatelet serotonin content [101]. The isolation method of easy-to-activate platelets can also influence the measured concentration as this monoamine is taken up from plasma, stored in dense granules of platelets, and released upon stimulation, contributing to an aggregative response [82]. Therefore, by having an effect on the degree of activation and degranulation, the choice of anticoagulant and centrifugation force can strongly influence serotonin concentrations [102]. This might be one reason for the inconsistent results (positive and negative ES in Table 1) for this parameter and great care should be taken if serotonin is considered as AD biomarker.

### Monoamine oxidase B

Another neuropharmacological drug target frequently studied in platelets is the neurotransmitter-degrading enzyme monoamine oxidase (Mao, EC 1.4.3.4). The existence of Mao in platelets was first reported in 1964 [103]. This enzyme exists in two isoforms, Mao-A and Mao-B, which are encoded by two genes with exactly the same exon-intron pattern, implying evolvement from a common ancestral gene. Differences do exist though in their primary structures and tissue-specific expression patterns, substrate preferences, and inhibitor sensitivities [104]. Mao-B is a mitochondrial membrane protein that catalyses oxidative deamination of monoamines, including phenethylamine and the neurotransmitters dopamine and aforementioned serotonin. Human platelets exclusively express Mao-B with the same amino acid sequence as brain Mao-B [105]. This enzyme has been proposed to be involved in ageing, as well as the pathogenesis of AD and Parkinson’s disease (PD) through increased generation of reactive oxygen species (ROS) and neurotoxic aldehyde catabolites [106]. The observation that Mao-B activities in brain and platelets correlate positively with ageing [107] has led researchers to suggest a systemic alteration of this potential neurological biomarker. In the course of AD, Mao-B activity was elevated in the temporal lobe and white matter as compared to age-matched controls [108]. These profiles were confirmed at the mRNA level in brain tissue, signifying that the increased activity arises from an enhanced level of transcription and a higher Mao-B concentration rather than from post-translational mechanisms [109]. Though, intraneuronal increase in Mao-B activity in both AD and ageing has been doubted [110, 111] with the hypothesis that because of higher constitutive Mao-B expression by glial cells, elevated brain Mao-B levels may be a consequence of gliosis during AD [108, 110].

Already in 1980, the finding by Adolfsson *et al.* of a similar increase in Mao-B activity in brain and platelets of AD patients suggested to use platelets as a peripheral diagnostic tool for AD [60]. Higher Mao-B activity in patient samples could only be detected with the Mao-B-specific substrate phenethylamine. These AD-related findings were subsequently confirmed in at least 12 clinical studies from different research groups [57, 59, 73, 112–120]. Five other studies, however, reported no AD-related activity increase [58, 61, 73, 99, 121]. Importantly, instead of phenethylamine, three of these studies used the substrates tyramine or kynuramine [61, 73, 99], both converted by Mao-A and Mao-B [122]. In a recent study, Mao-B activity quantified by kynuramine has been found to be significantly lower in late stage AD patients as compared to healthy controls, whereas in early and middle stage AD Mao-B activity was not changed significantly [99]. This finding stands in strong contradiction to the majority of previous studies, where most AD patients also suffered from moderate (mini–mental state examination (MMSE) score <20) to severe (MMSE score <10) dementia (unless otherwise specified; Table 1). Curiously, there does also not seem to be an agreement in the conclusions drawn by this group, since they additionally demonstrated a significant increase in Mao-B activity in a considerably larger AD patient cohort with an average MMSE of 18.9 [98].

In an unbiased proteome analysis, our laboratory found a predominant elevation in Mao-B expression in platelets from AD patients which strongly correlated with the enzymatic activity [62]. Nevertheless, concentration of Mao-B also increased with age (55 to 104 years) in healthy subjects. Centenarians exhibited platelet Mao-B levels comparably high to those of AD platelets [62], potentially reflecting the age-dependent cognitive decline indicated by lower MMSE scores. This suggests an age-related increase in Mao-B expression that is more pronounced in AD patients.

Despite a number of studies about excessive platelet and brain Mao-B activity in AD, little is known about the molecular causes underlying these changes. Platelet Mao-B activity is strongly influenced by lifestyle factors such as nutrition, alcohol, and smoking. Norharman, a compound of tobacco smoke, is a specific Mao-B inhibitor [123]. Paradoxically, it was observed that Mao-B inhibition in smokers was accompanied by elevated enzyme concentration in platelets [124]. Smoking induced a hypomethylation of the Mao-B promoter, followed by an increased Mao-B protein expression [124]. Since high homocysteine, as well as low vitamin B_12_ and folate levels are associated with AD [125], homocysteine-lowering therapies based on these B-vitamins counteract the accelerated atrophy in brains of MCI patients [126]. In good agreement with these observations, a correlation between plasma vitamin B_12_ levels and platelet Mao-B activity was observed in dementia patients: the lower the vitamin levels, the higher the enzymatic activity. Following vitamin B_12_ supplementation, platelet Mao-B activity was significantly reduced in these patients to apparently normal levels [127]. Recently, we have shown that a high animal protein diet in healthy young adults decreased platelet Mao-B expression by 26% compared to controls fed with normal protein diet. This was accompanied by improved cognitive function and correlated with increased plasma vitamin B_12_ levels [128]. Since both increased Mao-B protein expression levels and enzymatic activity (determined with the substrate phenylethylamine) in AD patients have repeatedly been reported by different research groups (Table 1), Mao-B is one of the most reliable and promising AD biomarker candidates.

### Nitric oxide and oxidative stress

Nitric oxide (NO) produced by activated astrocytes is thought to contribute to neurodegenerative processes. Reaction of NO with oxygen radicals leads to formation of peroxynitrite (ONOO^−^). This unstable structural nitrate isomer generates cytotoxic species which oxidise and nitrate proteins. Increased levels of nitrated proteins have been reported in AD brain and CSF [129]. Platelet NO production, as well as ONOO^−^ levels were evaluated in a large AD study cohort and significantly increased in afflicted individuals [44] with one of the highest ES of 6.3 (Table 1). In a subsequent study, elevated NO and ONOO^−^ production was more pronounced in male than female AD patients, but generally higher in males [45].

Defects in the mitochondrion’s electron transport chain are the main source of ROS, the central element of the free radical theory of ageing [130]. Given that ageing is the main risk factor for AD, this may also have an impact on the pathology of AD. Platelets have a high number of mitochondria what makes them attractive to study AD-related systemic malfunctions of the electron transport system. Investigations have revealed that both in isolated platelet and hippocampal mitochondria cytochrome c oxidase (complex IV of the respiratory chain) activity was significantly lower in AD patients as compared to controls [50]. Different research teams have confirmed this finding consistently [49, 51–53, 131, 132]. While one of these studies additionally found reduced adenosine triphosphate (ATP) concentrations in AD platelets [49], another investigation reported no significant differences between platelet ATP levels of patient and control groups [133]. Increased ROS levels in AD platelets have been detected [49], just as an enhanced blood lactate concentration correlating inversely with diminished platelet cytochrome c oxidase activity [51]. Similar findings in adult children of AD-affected mothers imply an exclusively maternal heredity of this biomarker [134]. Taken together, most results point towards the importance of increased oxidative stress in AD. Whether any of the indicators could be used as biomarker remains to be elucidated.

### Inflammatory mediators

In the periphery of amyloid plaques and NFTs, pro-inflammatory molecules are highly expressed [135]. One enzyme synthesised by platelets after an inflammatory stimulus is cyclooxygenase-2 (COX-2) that in turn is responsible for the production of prostaglandins. Several studies indicate that treatment with non-steroidal anti-inflammatory drugs and COX-2 inhibitors may reduce the risk of developing AD [136]. Indeed, Western blot analysis revealed a 50% increase in platelet COX-2 in MCI and a 25% increase in AD patients [137]. COX-2 expression profiles in platelets could indicate which patient groups may benefit from a COX-2 inhibitor therapy. Further details can be found in a recent review on inflammatory AD biomarkers in platelets [138].

### Amyloid precursor protein

One of the first milestones in understanding the pathogenesis of AD dates back to 1985, when cerebral Aβ deposits in senile and neuritic plaques were recognised as playing a central role [139, 140]. Amyloid plaques are formed by aggregation of Aβ peptides produced by proteolytic cleavage of APP. This single-pass type I membrane protein can be N-terminally hydrolysed by two alternative, differently initiated routes. In the non-amyloidogenic pathway, α-secretase cleaves APP first, releasing the neuro-protective, soluble fragment sAPP-α. In the amyloidogenic pathway, though, β-secretase is the first enzyme to cleave and the resulting soluble fragment is sAPP-β. The remaining carboxyl-terminal fragment derivatives are subsequently cut by γ-secretase to generate either a 3 kDa product or Aβ, respectively. Accordingly, when α-secretase cleaves, Aβ is not produced [141]. It is hypothesised (but still under debate [142]) that Aβ is a causative molecule of AD by inducing neuronal cell death and concomitant disturbance of synaptic function.

Platelets are equipped with α-, β-, and γ-secretases [143]. Studies have shown a reduction of α-secretase protein level in platelets of early stage AD patients [30, 144], in line with attenuated release of sAPP-α from thrombin-activated AD platelets [145]. In addition, an increased activity of β-secretase was indirectly shown by a decreased ratio of its 37/56 kDa fragments from two different groups [143, 144]. Another group determined it directly by an enzymatic assay with a 17% elevation in activity in AD platelets [41] and with an even more pronounced 24% upregulation in MCI platelets [42]. The concentration of β-secretase cleavage products was also found to be significantly increased [145]. On the other hand, the enzymatic activity did not correlate with MMSE scores, signifying that it might be a primary pathophysiological sign and may predict disease onset [41]. A recent study has confirmed altered soluble fragment ratios: while sAPP-α level were unchanged in both MCI and AD patients, there was a strong increase in sAPP-β level. Therefore, an ELISA-based assay to detect this altered β-fragment released by platelets incubated with recombinant BACE1 might be used as diagnostic screening tool [146].

Alternative splicing generates several APP mRNAs with the three major isoforms being APP695, APP751, and APP770 [141]. While the two longer forms (APP751 and APP770) possess a Kunitz-type serine protease inhibitor domain and are found in most tissues, the shorter APP695 is predominantly expressed in neurons [147]. Analysis of brain biopsies is difficult because of the mixture of cells such as astrocytes, glia cells, and neurons. Additionally, protein profiles may be influenced by gliosis (generally occurring in AD), masking neuron-specific expression changes. Microdissection studies would specifically show which proteome changes originate from a single cell type but only few have been conducted from AD brain so far. Therefore, scant and conflicting data are available on abundance and processing of the Aβ parent molecule in AD-affected brains [148–151]: APP751 and APP770 isoform patterns were found to be unchanged but concentrations of total APP and fragment APP695 decreased on mRNA level [150] and protein level [151] with an increase of the Aβ peptide level in samples of the frontal cortex of AD cases [151]. Due to these confusing data on neuronal APP isoform abundance, platelets may be an interesting alternative sample material. At this point it should be mentioned that it is assumed that generation of Aβ is boosted in EOFAD, whereas clearance of Aβ may be diminished in LOAD [152]. Since above mentioned alterations of APP processing and isoform abundance are mainly found in LOAD patients, this suggests that not only clearance of Aβ is affected in LOAD.

In platelets, the two prevalent isoforms are APP751 and APP770 though APP695 is also present [153]. This fact may have significance in haemostasis, since the Kunitz-type domain inhibits certain blood coagulation factors [154–156]. Intraplatelet localisation of the numerous APP isoforms is also different: full-length protein (140–150 kDa) is plasma membrane-bound, the predominant 100–130 kDa species (C-terminally truncated forms in activated platelets) are located in α-granule membranes [157]. APP metabolism has been found to be specifically altered in platelets of LOAD patients: a large number of publications from different groups report a decreased ratio of the two major platelet APP isoforms (130 kDa/110 kDa) in AD patients [26, 33, 35, 36, 39, 143, 158, 159]. One of the studies could also distinguish AD from PD and stroke patients since these values equalled those of controls [158]. Still others correlated the reduced ratio with disease progression [160]. Thus, despite huge variation in the reported values, APP ratio is still the most reproducible and promising AD biomarker in platelets to date (Table 1).

Interesting in this context is a newly identified C-terminally-truncated 115 kDa APP isoform which is not glycosylated and non-releasable upon platelet activation [32]. Experiments of this laboratory suggest that this fragment could represent an easily detectable diagnostic marker as it significantly and inversely correlated with reduced 130/110 kDa isoform ratios of AD patients. From a therapeutic perspective, the acetylcholine (ACh) esterase inhibitor donepezil was found to restore APP metabolism [145] and alter APP ratios in AD patients [40]. At the same time, statin-lowered cholesterol levels inversely correlated with increased APP ratios [158], indicating great potential of the APP isoform ratio or the 115 kDa fragment as a prognostic and surrogate marker for medication efficacy.

Since several smaller isoforms are produced from 130 kDa APP upon platelet activation [161], it can be possible that APP ratios in AD are due to altered platelet reactivity. This might have an important influence on APP biomarker studies as platelets are easily activated during sample collection. On top of that, anticoagulants strongly affect platelet activation and degranulation, as demonstrated previously [28]. Great care is therefore essential to avoid activation during venipuncture, blood collection, and platelet isolation. CTAD blood tubes [162] (containing citrate, theophylline, adenosine, and dipyridamol) are most suitable, as indicated by strongly reduced levels of plasma platelet factor 4 compared to those measured from EDTA or citrate blood tubes [28].

### Membrane fluidity and cholesterol

There are indications that low membrane cholesterol levels have a considerable impact on the pathogenesis of AD [163]. Cholesterol is the major lipid constituent of biological membranes and plays a key role in defining their physical state by regulating fluidity in a concentration-dependent manner [164]. An example was provided in a study demonstrating that an initial decrease in cholesterol content of total brain lipid extracts by approximately 5% *in vitro* also reduced membrane fluidity, whereas further decrease in cholesterol increased fluidity again [165]. Moreover, this study could demonstrate that addition of Aβ_1–40_ to brain lipid membranes resulted in diminished vesicle fluidity, thereby linking the amyloidogenic pathway with altered membrane fluidity. In line are findings that Aβ aggregates can disturb the structure of brain membranes and that membranes derived from AD-affected hippocampi had lower fluidity [166]. This together with lower cholesterol levels in brain preparations of AD patients challenge the application of cholesterol-lowering statins as therapeutic agents [167].

Literature on AD accompanying changes of platelet membrane fluidity is rather inconsistent. Platelet membrane cholesterol content has been found to be lower in cognitively impaired subjects and β-secretase activity correlated bimodally with these levels: when concentrations were below 50 pmol cholesterol/μg membrane protein, the correlation was negative, above this threshold it was positive [168]. Additionally, this study identified elevated cholesterol levels in platelet membranes of statin-treated versus untreated subjects. Another study has detected reduced fluidity in platelet membranes from AD patients; this effect was even more pronounced in males [45]. Similarly, lower fluidity has been detected in membranes of sub-mitochondrial particles of AD platelets [66]. On the other hand, only insignificant differences in this parameter between LOAD patients and controls have been reported [169]. Despite a weak correlation with the 130 kDa/110 kDa APP ratio, platelet membrane fluidity differed only minimally between AD, MCI, and controls [37]. Others have even reported results pointing to the opposite direction: elevated fluidity in AD platelet membranes [64, 170, 171] and intracellular membranes [172]. This inconsistency on the relationship of Aβ, cholesterol, and AD has been reviewed with the outcome that more data are needed before a definitive conclusion can be drawn on the connection of membrane fluidity and AD pathogenesis [173]. Since AD is an age-related disease, it needs to be emphasised that membrane fluidity is also reduced during ageing, a decline less pronounced in centenarians [174].

Na^+^/K^+^-ATPase is another indicator of membrane functionality as the physicochemical properties of the microenvironment have great influence on the activity of this integral membrane protein. The activity of this sodium-potassium pump has been found to be reduced in platelets of AD patients [44]. In agreement are results of significantly diminished levels of both Na^+^/K^+^-ATPase enzymatic activity and protein expression in AD brains [175]. A further study compared its activity in platelets from male and female AD patients as well as control subjects. While enzyme activity was generally higher in females, an AD-related decrease persisted within both gender groups [45]. In summary, these results indicate that Na^+^/K^+^-ATPase activity could be a promising candidate in AD diagnosis with a high ES of −6 (Table 1).

### Phospholipase A2

Another enzyme family involved in membrane physiology are the phospholipases A2 (PLA2, EC 3.1.1.4) which play an important role especially in lipid metabolism. Catalysing the hydrolysis of membrane glycerophospholipid ester bonds, free fatty acids (e.g. arachidonic acid) and lysophospholipids are generated [176, 177]. Their exact role in neurodegeneration is still to be explored as earlier data showed ambiguity regarding the consequences of altered PLA2 activity. On the one hand, studies have indicated that increased PLA2 activity alters membrane integrity which (especially via Ca^2+^-induced lipolysis) ultimately leads to cell structure disruption and thus to neurodegeneration [178]. On the other hand, in animal experiments inhibition of PLA2 impaired learning and spatial memory, similar signs occurring in the earliest phases of AD [179]. Additionally, PLA2 inhibition led to reduced fatty acyl chain flexibility, linking PLA2 with AD via altered membrane fluidity [180]. In line, membrane phospholipid metabolism has been described to be reduced in the prefrontal cortex of mildly and moderately demented AD patients [181]. These findings of PLA2-dependent reduction in membrane hydrophobic core fluidity correspond with the recent observation of diminished membrane fluidity in platelets of AD patients [45]. Moreover, PLA2 activity was significantly reduced in parietal and frontal cortices of AD patients; this enzyme deficiency could be connected with earlier onset and severity of the disease, as well as a worse outcome [182]. A further hint of a link between PLA2 and AD was provided by differentially processed and secreted APP after inhibition or activation of PLA2 [183]. An increase in both sAPPα secretion and membrane fluidity, as well as a decrease in Aβ_1–42_ levels was detected when neuronal cells were exposed to sPLA2 [184].

The search for peripheral surrogates of cerebral changes in AD encouraged several groups to investigate PLA2 activity in platelet membranes. In 1996, a study demonstrated significant reduction in enzymatic PLA2 activity in platelets of AD patients [67]. In a subsequent publication, this group reported a correlation of lower platelet PLA2 activity in MCI and AD patients with severity of cognitive decline. Accordingly, MCI individuals had mean PLA2 activity levels between those of AD patients and controls [68]. After a one-month long cognitive training period, platelet PLA2 activity increased in healthy elderly subjects [185]. In contrast stands a significantly increased activity of PLA2 in platelets of AD patients reported by another team [69]. They assumed that the differences of these results may be due to different severity of the disease since their AD patients were at an earlier stage. Finally, a 4-year follow-up study of MCI subjects detected that patients with a low baseline platelet PLA2 activity had a higher risk of progressing to AD, suggesting that low platelet PLA2 activity may be an AD risk marker in such patients [70]. As indicated in Table 1, only few data sets originating from different publications are available. Together with the low sample numbers and inconsistent ES, we would not recommend PLA2 as trustworthy AD platelet biomarker until other researchers confirm a reduced platelet PLA2 expression in AD and MCI patients.

### Tau

A second hallmark of AD is represented by intracellular NFT that consist of hyperphosphorylated tau protein [186, 187]. Tau is mostly found in axons where it attaches to and stabilizes microtubules, crucial for anterograde and retrograde axonal transport. Hyperphosphorylation of tau causes it to dissociate from microtubules, thereby greatly reducing their stability and ultimately leading to cell death. Free hyperphosphorylated tau tends to aggregate into helical filaments which result in formation of the aforementioned NFT [188, 189].

Apart from hyperphosphorylation, aberrations in tau splicing are further regulations directly causing neurodegenerative diseases [190]. High molecular weight tau protein (130 kDa) was detected in tangles of AD brains already in 1992 [191]. Several years later, a 110 kDa tau variant was also found in peripheral tissues such as human oral epithelium [192] and rat muscle [193]. Expression of tau protein in human platelets has been reported [56] together with an elevated ratio of high molecular weight (>80 kDa) oligomeric isoform variants to monomeric tau in AD patients [24]. In a follow-up study, this group identified tau modifications also in healthy subjects, without any age dependency. Nevertheless, the above described ratio seemed to correlate with the cognitive status of AD patients [27]. Other studies analysed platelet tau quantities in AD patients and control subjects but could not detect any disease-specific differences [25, 56]. However, C-terminal end tau protein levels of MCI subjects were significantly different from normal ones. Additionally, they detected elevated total tau levels in older AD patients as compared to both younger AD patients and healthy controls, concluding that tau might be a diagnostic marker for the detection of the onset of the disease [25]. Together, results on platelet tau as AD biomarker are sparse, unsettled, and originate from two research groups only. Thus, interpretation should be done with great care and more data are required to define its diagnostic value, ideally by conducting clinical studies.

### Glycogen synthase kinase 3β

One of the most important tau kinases in neurons is glycogen synthase kinase 3β (GSK3β) which is suspected to play a central role in AD [194]. Its activity has been found to be increased in specific regions of the AD brain [195–197], thereby promoting hyperphosphorylation of tau and formation of NFTs. Normally, GSK3β is constitutively expressed in all cells and primarily inhibited via phosphorylation of serine-9 [198]. Lithium can increase GSK3β phosphorylation indirectly [199], thereby reducing its tau hyperphosphorylation activity and stabilising cognitive ability in AD patients [200]. Comparison of the ratio of Ser-9-phosphorylated GSK3β versus total GSK3β in platelets of AD patients and controls revealed a significant reduction in AD and MCI samples, indicating seriously enhanced enzyme activity [54]. This ratio correlated with cognitive scores and might therefore represent an interesting diagnostic target in platelets. Nevertheless, these findings need to be reproduced by others before the diagnostic value can be defined.

## Conclusion

Due to increased ageing of the world population and concomitantly elevated prevalence of AD, a vast number of publications have been produced during the last decades, deepening our understanding about AD and accompanying molecular changes. Nevertheless, to date there is no alternative for diagnosis of *definite* AD than postmortem brain autopsy. However, findings that blood platelets could serve as a source of efficient biomarkers to assist early AD detection are promising.

Here, we compiled AD platelet biomarker candidates with available information to calculate their Hedge ES as a measure for comparability in a tabular form (Table 1). AD-related alterations (and standard deviations) of various molecules and physicochemical platelet changes from different studies are quite heterogeneous and data therefore difficult to compare. In order to offer a more standardised factor, the Hedge ES is provided as useful tool to determine the separating power of a given biomarker. It is interesting to observe that highest ES were calculated for potential biomarkers that are indicators of either oxidative stress or membrane integrity. This supports the idea that inflammation and oxidative stress play crucial roles in the pathogenesis of AD. The quite large ES of sub-mitochondrial platelet membrane fluidity implies that these structures become extremely sensitive to oxidative stress and may be involved in initiating disease progression. At the same time, it is important to note that ROS production is considered to be a function of ageing, which in turn is the most significant risk factor of LOAD. However, the large effect sizes of NO metabolism and membrane fluidity together with changed Na+/K + −ATPase activity have only been shown by one laboratory so far [44] and need to be validated by other research facilities.

Conflicting data on some potential AD biomarkers are highlighted by inconsistent ES calculated for e.g. platelet membrane fluidity and serotonin concentration (Table 1). These contradictions are perhaps due to difficulties analysts face when examining heterogeneous and hard-to-identify target populations such as suspected AD patients and their appropriate controls. Furthermore, it is crucial to avoid activation in the case of platelets, thus the choice of both the anticoagulant and the protein extraction method can modify the outcome of any blood-based study. Complications also arise from the lack of standardised sample collection and isolation methods, which have a dramatic impact on the results. These latter shortcomings are not restricted to platelets either but generally apply to biomarker studies in all media, e.g. CSF or plasma, which have similar drawbacks. Accordingly, some of the less obvious candidates like membrane fluidity have been found to be both increased [170] and decreased [44] in AD patients. On the other hand, some of the molecular marker candidates indeed showed great reliability, such as decreased APP isoform ratio and APP-processing proteases ADAM10 and BACE1. Elevated levels of multimer tau, the second hallmark of AD, have been reported, though available data are limited to two publications from the same group [24, 27] and a contradictory report from another [25]. Mao-B, a further highly promising candidate, has uniformly been found to be up-regulated with quite stable ES, at least when the substrate phenethylamine was used. We suggest that Mao-B and APP isoform ratios are currently the two top AD biomarker candidates in platelets due to consistent results from several different groups.

Finally, we can conclude that platelets represent a promising peripheral surrogate to detect AD-related molecular changes and provide crucial data necessary for taking the next step towards development of a diagnostic and/or therapy-predictive tool for AD. However, as none of the individual markers described is powerful enough to meet the required levels of sensitivity and specificity for routine AD diagnosis [201], it may be useful to exploit several of these biomarker candidates contemporaneously. It remains to be seen whether a combination would be robust enough to expose molecular changes occurring at the early phase of AD so as to distinguish between healthy and affected individuals. Future work into AD platelet biomarkers might shift the focus towards a proteomic approach in order to identify the best combination of biomarkers with the intention of designing diagnostic multiplex devices.
